# Transformational leadership and employee AI usage: the role of perceived organizational support and competitive workplace climate

**DOI:** 10.3389/fpsyg.2025.1581337

**Published:** 2025-11-19

**Authors:** Hong-Yan Wang, Rui-Hong Liu, Lie Ao

**Affiliations:** College of Economics and Management, Hubei Polytechnic University, Huangshi, China

**Keywords:** transformational leadership, perceived organizational support, employee artificial intelligence usage, competitive workplace climate, social cognitive theory, moderated mediation model

## Abstract

**Introduction:**

Artificial intelligence (AI) has become integral to organizational transformation and daily management, making employee AI usage (AI-U) an increasingly prevalent phenomenon. However, despite its growing importance, little is known about how leadership and contextual factors shape employees’ usage of AI.

**Methods:**

Based on social cognitive theory, this study investigates the mediating role of perceived organizational support (POS) and the moderating effect of competitive workplace climate (CWC) in the relationship between transformational leadership (TL) and employee AI-U. Data were collected from 525 employees in China through an online survey and analyzed using hierarchical regression analysis and bootstrap methods.

**Results:**

The results revealed that TL positively predicts employee AI-U, and that POS partially mediates this relationship. Moreover, CWC significantly moderates the indirect effect, such that the mediating effect of POS is weakened in a high-level CWC.

**Discussion:**

These findings enrich the understanding of AI adoption from a social cognitive perspective and offer practical insights for fostering supportive organizational conditions conducive to AI application.

## 1 Introduction

Artificial intelligence (AI) refers to a suite of technologies that enable machines to perform tasks that typically require human intelligence (Arerkar, 2019; Tang et al., 2023). Since its inception in the late 1950s, the field of AI has evolved to produce a wide array of applications, from automated systems and robotics to advanced deep learning architectures. The global AI market is projected to grow at a compound annual rate of 37.3% from 2023 to 2030 (Grand View Research, 2023), reflecting its transformative impact across industries (Li et al., 2019). For example, a recent report indicates that 49% of U.S. enterprises already use ChatGPT, with another 30% planning to do so (Resume Builder, 2023). A global survey further shows that AI adoption in business functions rose from 55 to 78% within a year (Hanselman, 2025). However, scholars hold divergent views on AI’s organizational impact. Some emphasize its potential to enhance efficiency and effectiveness (Pillai and Sivathanu, 2020; Tang et al., 2022), while others warn of adverse outcomes like job displacement (Gursoy et al., 2023). Concerns are particularly acute regarding unsanctioned use in sensitive sectors (e.g., finance, healthcare, and education), where risks include data privacy breaches, compromised decision-making, and regulatory violations (Xue et al., 2023; Balogun et al., 2025). Despite the need for further exploration of AI’s effects, the potential for such negative outcomes has led a growing number of organizations to mandate sanctioned rather than unsanctioned AI use among employees. Consequently, this study narrows its focus to the context of sanctioned AI usage (AI-U), wherein such tools are formally approved and implemented by the organization.

These conflicting perspectives trigger varied employee responses to AI usage (AI-U). Some employees leverage it to improve efficiency, performance, and creativity (Shaikh et al., 2023; Tasgit et al., 2023; Verma and Singh, 2022). Others exhibit skepticism or resistance, leading to counterproductive behaviors, reduced commitment, or higher turnover intentions (Tasgit et al., 2023; Zhang et al., 2023). As AI becomes increasingly prevalent (Brynjolfsson and McAfee, 2017; Davenport and Ronanki, 2018), understanding how employees engage with it in daily work is essential.

AI-U is defined as the extent to which employees incorporate AI technologies into their daily tasks to perform functions intelligently (Tang et al., 2022). Common applications include using ChatGPT to compose emails, generate copy, translate texts, and write or debug code, etc. Since technologies possess social attributes (Liu et al., 2024) and users play a critical role in enacting their capabilities, identifying factors that motivate AI-U has gained academic and practical interest (Tang et al., 2023; Potinteu et al., 2023; Shaikh et al., 2023; Liu et al., 2024). While prior research has focused on individual-level antecedents like personality, trust, and risk perceptions (Park and Woo, 2022; Potinteu et al., 2023), broader contextual factors, especially leadership, remain underexplored. Leaders serve as key sources of social influence. Through observational learning (Bandura, 1986), employees infer valued behaviors by observing their leaders. Social cognitive theory (SCT) provides a useful framework for this dynamic, positing that human functioning arises from the interplay of personal, behavioral, and environmental factors (Bandura, 1986; Schunk and DiBenedetto, 2020). This theory moves beyond viewing individuals as passive recipients of technology, allowing us to examine how leadership—as an environmental factor—shapes employees’ cognitive perceptions (e.g., support), which in turn drive AI-U behavior. Although leaders are pivotal in AI adoption (Peifer et al., 2022; He et al., 2023; Liu et al., 2024; Mousavi et al., 2025), most studies address organizational-level implementation, with limited attention to how specific leadership styles, such as transformational leadership (TL), affect individual-level AI-U.

Transformational leadership (TL) describes leaders who inspire followers to transcend self-interest for the sake of the organization through vision, motivation, and intellectual stimulation (Bass, 1999; Reza, 2019). In digital transformation, TL can play a vital role in promoting positive AI adoption. As AI often requires extra effort to master (Potinteu et al., 2023) and its use may extend beyond formal role requirements, employees are likely to interpret leaders’ attitudes as signals of organizational expectations. Given that TL is value-neutral, its effects depend on the leader’s strategic focus; therefore, this study narrows its scope to contexts where leaders exhibit favorable attitudes toward sanctioned AI-U. Leaders who hold favorable attitudes toward policy-aligned AI adoption are better positioned to enhance employee awareness and engagement with these technologies (Shaikh et al., 2023). Specifically, when leaders exhibit favorable attitudes toward AI, they can articulate a compelling vision, model appropriate behaviors, and provide supportive feedback—all of which may enhance employee engagement with AI. Moreover, since leadership is often seen as representing the organization, such support may be perceived as organizational backing, strengthening employees’ confidence in using new technologies. In this context, perceived organizational support (POS) may serve as a mediating mechanism, helping to translate TL into employee AI-U behavior by fostering a supportive perceptual environment. However, this pathway lacks theoretical and empirical validation. Thus, this study seeks to address this issue by investigating the mediating role of POS in the relationship between TL and AI-U.

The influence of TL on employee AI-U is likely not uniform but contingent on contextual factors. SCT emphasizes that the impact of environmental cues is not uniform but is appraised and interpreted by individuals within their specific context (Schunk and DiBenedetto, 2020). A particularly relevant contextual factor is the competitive workplace climate (CWC), which refers to “an organizational environment where employees are compelled to assess their performance in comparison to others, leading to a sense of competition and pressure” (Wang et al., 2024, p. 7). CWC is embedded in an organization and takes the form of a shared culture and set of practices that shape the employees that it surrounds (Hofmann et al., 2003). These shared perceptions and behaviors subsequently form the basis for employees’ subjective evaluations of competition in their workplace. Such evaluations, in turn, may influence how individuals interpret and respond to competitive pressures, often reflecting broader cultural and institutional factors. Studies conducted in East Asian contexts (e.g., China and Korea) have shown a pronounced tendency toward intra-organizational competition (Yang, 2021). While organizations may foster competitive climates to drive performance (Markovich et al., 2021), CWC can function as a double-edged sword, enhancing performance on one hand (Ye et al., 2020; Wang et al., 2024) but also triggering negative outcomes such as workplace envy and cheating (Mohd Shamsudin et al., 2023). However, how CWC affects the relationship between TL and employee AI-U has not yet been explored; thus, this study aims to explore this topic.

This study supplements the extant literature on this topic in three ways. First, it extends existing work on the antecedents of AI-U by investigating the role of TL. While previous studies have emphasized individual-level factors (Park and Woo, 2022; Potinteu et al., 2023), this study highlights the importance of leadership style in shaping employees’ responses to AI-U. Second, it introduces POS as a mediator between TL and AI-U, offering a theoretical mechanism that explains how leadership behavior translates into employee behavior through perceptions of organizational support. Third, it examines the moderating effect of CWC, acknowledging that the efficacy of TL may vary under different organizational climates. Previous studies have indicated that CWC in the workplace has a double-edged sword effect (Ye et al., 2020; Mohd Shamsudin et al., 2023; Wang et al., 2024). However, until recently, no research has focused on the connections among TL, CWC, and AI-U. This study not only enriches the literature on AI-U and leadership but also offers a more nuanced understanding of how environmental factors interact with leadership behaviors.

On the basis of SCT, this research advances the understanding of employees’ attitudes and behavioral responses to AI by exploring how TL influences employees’ AI-U. The remainder of the paper is structured as follows: The next section reviews relevant literature and develops hypotheses. Then, the methodological approach—including data collection, measurement, and analytical strategies—is described. Subsequently, empirical results are presented. The paper concludes with a discussion of theoretical and practical implications, limitations, and future research directions.

## 2 Literature review and theoretical hypotheses

### 2.1 Transformational leadership (TL) and artificial intelligence usage (AI-U)

Transformational leadership (TL), one of the most influential leadership styles in the management literature (Yukl, 2012; Guerrero et al., 2017), emphasizes emotions, beliefs, and values (Bass, 1999). It comprises four components: idealized influence, inspirational motivation, intellectual stimulation, and individualized consideration (Bass, 1999; Reza, 2019). TL has been widely shown to positively influence employees’ attitudes and behaviors, including job performance (Bakker et al., 2023), change management (Bagga et al., 2023), innovative behavior (Jun and Lee, 2023), well-being (Gaur, 2023), and digital transformation (Philip, 2021). Despite this extensive research, its role in shaping employee AI usage (AI-U) remains underexplored. In the rapidly evolving AI era, organizations must adapt to unprecedented environmental shifts to ensure survival and sustain development (Mikalef et al., 2021). The proliferation of AI technologies presents both opportunities and imperatives for organizational change, requiring leaders who can effectively guide digital transformation efforts (Philip, 2021). In this context, TL is well-suited to this context, as it involves articulating a compelling vision, recognizing external demands, and shaping adaptive organizational responses (Ghamrawi, 2013; Mikalef et al., 2021; Bagga et al., 2023). By enhancing employee awareness and engagement with the technology (Shaikh et al., 2023) and developing of employees’ capacity to effectively adopt technological resources such as sanctioned AI tools and resources (Bagga et al., 2023; Jun and Lee, 2023), TL strengthens the organization’s ability to integrate emerging technologies into its core practices.

It is important to note that AI in the workplace serves not only as an instrument of performing work tasks but also as a collaborative “colleague” (Tang et al., 2022). Yet, the realization of such roles to some extent depends on a user’s response to AI-U in the workplace. Prior research on employee AI-U has primarily focused on domain-specific contexts such as education (Abbas et al., 2024), customer service (Xu et al., 2020), healthcare (Mousavi et al., 2025), and public service (Gesk and Leyer, 2022). Recent studies have increasingly investigated individual-level determinants, including personality traits (Park and Woo, 2022) and perceptions of AI (Potinteu et al., 2023; Mousavi et al., 2025). However, situational factors particularly leadership remain underexplored in shaping AI-U. According to SCT, the effectiveness of leadership influence depends significantly on the social persuasions and supportive environment leaders create. Transformational leadership articulate a compelling vision for AI, model its use, and provide supportive feedback. Such supportive actions fostered by TL may encourage employee AI-U, especially since transformational leaders tend to be sensitive to external dynamics and skilled at integrating new technologies (Bagga et al., 2023; Jun and Lee, 2023). Furthermore, by alleviating routine task burdens and improving information-processing efficiency (Tang et al., 2022), AI can enhance work performance. Consequently, employees under TL may be more inclined to use AI in their daily work. Moreover, given that effective AI-U requires substantial learning (Potinteu et al., 2023), TL’s emphasis on self-development and adaptability (Shriberg and Shriberg, 2011) may further encourage the necessary skill acquisition, thereby promoting active AI-U. Based on the above reasoning, we propose that TL, particularly when leaders hold favorable attitudes toward policy-aligned AI adoption, enhances employees’ AI-U in the workplace. The hypothesis is as follows:

*H1:* TL positively enhances employees’ AI-U in the workplace.

### 2.2 Mediating role of perceived organizational support (POS)

Leaders’ acceptance of and support for AI are crucial to successful AI implementation within organizations (Peifer et al., 2022; He et al., 2023; Liu et al., 2024). We propose that perceived organizational support (POS) is the key mediating factor in TL affecting employee AI-U. POS refers to employees’ “beliefs concerning the extent to which the organization values their contribution and cares about their well-being” (Eisenberger et al., 1986, p. 501), constituting a form of psychological agreement between employee and organization (Aselage and Eisenberger, 2003). It represents a resource that provides material and emotional support (e.g., recognition, appreciation, and rewards) which fosters favorable employee attitudes and behaviors.

From the perspective of SCT, leadership constitutes a critical environmental factor influencing employee cognition and behavior. TL emphasizes rewards and intrinsic motivation (Kurtessis et al., 2017; Subs, 2019), which employees interpret as indicators of organizational support, thereby enhancing POS (Eisenberger et al., 1986). Prior studies confirm that TL significantly strengthens POS (Kurtessis et al., 2017; Sahban, 2019). Transformational leadership excel in navigating uncertain external environments and establishing higher standards and challenges (Suifan et al., 2018), and encourage their followers to seek new opportunities and new possibilities, thereby promoting their growth and development (Stinglhamber et al., 2015). Furthermore, these leaders also may articulate a compelling vision for AI, model its use, and provide supportive feedback. Since leadership is often perceived as representing the organization, these actions are interpreted by employees as strong indicators of organizational support.

However, despite established knowledge of the TL–POS relationship, little is known about how POS, in turn, influences emerging technology adoption behaviors among employees, particularly regarding AI-U. As the growing role of AI in enhancing organizational performance (Pillai and Sivathanu, 2020; Shaikh et al., 2023), the adoption of AI by employees has emerged as a primary concern for many organizations. From an SCT standpoint, POS constitutes a critical cognitive appraisal of the environment- a belief that the organization provides the necessary resources and support for successful adaptation. Employees who perceive strong organizational support are more likely to identify with organizational goals and engage in behaviors that align with those objectives—such as adopting sanctioned AI to improve performance (Suifan et al., 2018). Consequently, POS may serve as a vital mediating mechanism that translates leadership influence into the positive cognitive appraisals necessary for behavioral change. But to our knowledge, the mediating mechanism through which TL cultivates AI-U via POS has not been empirically established. Thus, the following hypotheses are proposed:

*H2a:* TL positively impacts employee POS.

*H2b:* POS positively impacts employee AI-U.

*H2c:* POS positively mediates the relationship between TL and AI-U.

### 2.3 The moderating role of a competitive workplace climate (CWC)

Leadership is inherently embedded within specific social context (Wisse et al., 2019), and technology is recognized as carrying social attributes (Liu et al., 2024). Consequently, investigating organizational contextual variables that may influence the relationship between TL and employee AI-U is critical. Among these factors, competitive work climate (CWC) represents a pervasive organizational phenomenon (Wang et al., 2018), often reflecting external competitive pressures that permeate internal operations. Scholarly attention has increasingly focused on CWC as a significant factor influencing employee attitudes and behaviors (e.g., Murtza and Rasheed, 2023; Wang et al., 2024). Originally conceptualized by Kohn (1992), competitive psychological climate captures employees’ subjective perceptions of win-lose dynamics within the work environment. Previous research suggests that CWC exerts a dual influence on employee outcomes, enhancing initiative, performance (Markovich et al., 2021; Ye et al., 2020; Wang et al., 2024), creativity (Nnadozie et al., 2019), service quality, and teamwork (Itani and Chaker, 2022), yet also potentially triggering negative outcomes including workplace envy, knowledge hiding (Mohd Shamsudin et al., 2023; Murtza and Rasheed, 2023), and cheating behaviors (Rai and Kim, 2023). However, little is known about how CWC moderates the relationship between leadership styles and AI usage behaviors. In this study, we propose that CWC triggers two distinct psychological mechanisms that operate in parallel, thereby differentially moderating the relationships among TL, POS, and AI-U.

TL promote change and innovation, encourage employees to challenge existing practices, explore new solutions, and capitalize on intrinsic and extrinsic incentives—including competition (Subs, 2019). Meanwhile, AI technologies become increasingly widespread in organizations, the leader exhibit increasingly high expectation thresholds for employees, and competitive pressure among employees may increase continually (Gim et al., 2015). In highly competitive climates, employees perceive that rewards and career advancement depend critically on leaders’ evaluations. This perception heightens their sensitivity to transformational leaders’ support and guidance, fostering a mechanism of instrumental reliance. Under these conditions, employees are more likely to proactively adopt performance-enhancing tools—such as AI—to gain a competitive edge and align with leader-endorsed initiatives (Wisse et al., 2019). Consequently, the positive influence of TL on AI-U is likely to be amplified under high CWC. In contrast, in low-competition environments, the motivational link between TL and specific, tool-oriented behaviors like AI-U may be less pronounced. Hence, this study posits the following hypothesis:

*H3a:* CWC positively moderates the positive relationship between TL and AI-U.

Competition is often regarded as an effective mechanism for motivating team members to pursue organizational objectives, but it is inherently a confrontational social dynamic. While CWC can sharpen employees’ focus on the leader as a resource gatekeeper (Ghamrawi, 2013), it simultaneously fosters a zero-sum perception of the workplace that may undermine broader perceptions of organizational support. Under high CWC, employees are more likely to attribute reward allocation and support primarily to the immediate leader’s comparative evaluations rather than to fair, systemic organizational policies. This attributional pattern fosters a “leader-first” orientation, wherein supervisory approval is prioritized, but the sense of belonging to a supportive collective organization is eroded. Consequently, even supportive actions from transformational leaders—who act as organizational agents (Ghamrawi, 2013)—may be attributed to the leader individually, weakening the translation of TL into a generalized POS. In low-CWC settings, however, transformational leaders are better able to foster collective interests and encourage employees to transcend self-centered goals (Meng et al., 2022), thereby strengthening the TL-POS relationship. Accordingly, we propose the following hypothesis:

*H3b:* CWC negatively moderates the relationship between TL and POS.

On the basis of the preceding hypotheses, we propose a moderated mediation hypothesis; namely, a high level of CWC weakens the indirect effect of TL on employee AI-U via POS. In contrast, a low level of CWC strengthens the indirect effect of TL on employee AI-U via POS. Therefore, we propose the following hypothesis:

*H3c:* CWC negatively moderates the indirect effect of TL on AI-U via POS.

**Figure 1:**
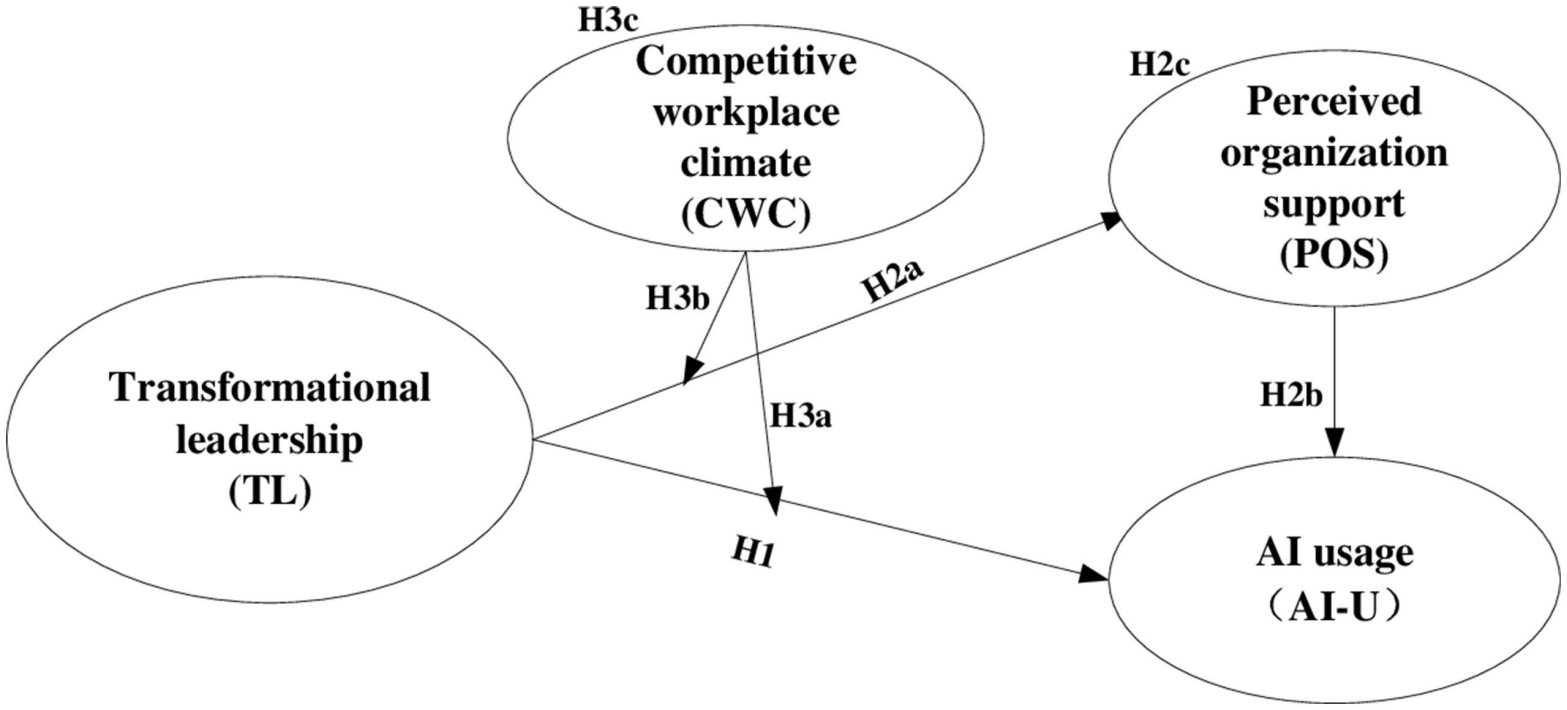
The theoretical hypothesis model. TL, Transformational Leadership; POS, Perceived Organizational Support; CWC, Competitive Workplace Climate.

Thus, on the basis of these theoretical foundations, we propose the theoretical framework used in this research, which is illustrated in Figure 1.

## 3 Methodology

### 3.1 Participants and procedure

We recruited participants online from the WJX platform^1^ from December 2023 to April 2024 in China. Compared with other sampling approaches, network survey platforms offer more diverse sample pools and thus facilitate the development of flexible questionnaire designs and the collection of high-quality data (Peer et al., 2017). All the participants provided informed consent via an electronic consent form and signature for participation, and they were assured that their responses were anonymous.

Our research materials and questionnaires are presented on the WJX platform. First, the participants were required to read a text pertaining to AI, including a description of the concept of AI, alongside several examples of the use of AI to perform work; for example, “an online salesperson uses algorithm-based systems to screen target customers and formulate personalized sale plans.” Second, the participants were asked to answer two screening questions in sequence. (1) The first screening question was a single-choice question: “Do you use AI to perform your work?” The participants who answered “yes” proceeded to the second screening question, whereas for those who answered “no,” the questionnaire automatically ended. (2) The second screening question focused on the following statement: “Please briefly describe your job position and the ways in which you use AI to perform your work.” A research assistant who was highly familiar with the definitions and characteristics of AI-U to evaluate the participants’ answers to the second screening question and whose evaluation criteria were based on the AI-U definition from Tang et al. (2022) emphasized the intelligent collaboration extent of AI in employees’ job tasks. Third, on the basis of the screening results of the second step, the participants were required to complete a formal questionnaire in which respondents’ demographic attributes (sex, age, education, work tenure, industry) and core variable measurements (TL, CWC, POS, and AI-U) were described. The formal survey questionnaire was completed in two stages separated by a two-week interval. Information pertaining to participants’ demographic characteristics, TL and CWC was collected in the first stage. The second-stage survey was implemented 2 weeks after the first stage with the same participants who responded to the first survey; at this time, information regarding participants’ POS and AI-U was collected. Finally, participants who completed the formal questionnaire were paid 10 RMB as compensation.

A total of 850 participants participated in the survey. In response to the first screening question, 212 participants answered “No,” thereby automatically ending the questionnaire. In response to the second screening question, 68 participants whose answers did not meet the evaluation criteria were excluded. In total, 570 individuals met the requirements stipulated by the statement question; these individuals were subsequently invited to participate in the formal survey.

After incomplete, invalid questionnaires were eliminated from the formal survey, the final effective sample size was 525, and the effective recovery rate was 92.00%. Among these 525 participants, 72.6% were female, while 68.4% were married; their average age was 28.5 years. In terms of education, participants who had obtained a college or associate degree or lower accounted for 2.5% of the sample, those who had obtained a bachelor’ s degree accounted for 63.2%, and those who had obtained a master’ s degree or higher accounted for 34.3%. With respect to participants’ workplace tenure (in years), 26.9% of the respondents had been in their current position for 5 years or fewer, 43.4% had a tenure of 5–10 years, 20.4% had a tenure of 10–15 years, and 9.3% had a tenure of 15 years or more. Participants were primarily employed in the technology, education, and service sectors, which comprised 85% of the sample. The remaining 15% worked in other industries.

### 3.2 Measurement

The scales used in this paper have been well established and are commonly used in the literature. The standard back-translation procedure was used to increase the accuracy of the translation (Brislin, 1980), and the questionnaires were translated into Mandarin Chinese from English. In addition to the team members, we invited two researchers who are experts in the study field of organization management digitization and two managers engaging in human resource management and technical work in the company. To improve the accuracy of questionnaire information and the effectiveness of completing the questionnaire, five full-time employees were also invited to check the questionnaire design and content. The items included in the questionnaires were scored on five-point Likert scales ranging from 1 (strongly disagree) to 5 (strongly agree).

Transformational leadership (TL). Participants were asked to evaluate the transformational leadership behaviors of their direct supervisors using the scale by Carless et al. (2000). This scale has been examined and demonstrated a reliability of 0.884 (Gaur, 2023). It contains seven items: vision, staff development, supportive leadership, empowerment, innovative thinking, leading by example, and charisma. Example items include “communicates a clear and positive vision of the future” and “gives encouragement and recognition to staff.” The overall Cronbach’s alpha coefficient for TL in this context was 0.819.

Artificial Intelligence Usage (AI-U). Daily AI-U was measured via a 3-item scale (Medcof, 1996). This scale has been used to evaluate employee AI-U in Chinese contexts and has good reliability (Cronbach’s alpha coefficient = 0.75; Mu et al., 2023). Sample items include “I worked with AI to make major work decisions,” “I used artificial intelligence to perform most of my job functions,” and “I spent most of my time working with artificial intelligence.” The Cronbach’s alpha coefficient for this scale in this context was 0.809.

Perceived Organizational Support (POS). POS was measured via an 8-item scale (Eisenberger et al., 1997). This scale has been examined and demonstrated a reliability of 0.88 (Maan et al., 2020). Sample items include “my organization cares about my opinions” and “my organization takes pride in my accomplishments at work.” The Cronbach’s alpha coefficient for this scale in this context was 0.842.

Competitive Workplace Climate (CWC). CWC was measured to assess employees’ perception regarding the intensity of competitive climate within the organization via the 6-item scale developed by Zhu et al. (2016). This scale has been used to evaluate employee perceptived CWC in Chinese contexts and has good reliability (Cronbach’s alpha coefficient = 0.843; Wang and Yin, 2021). Sample items include “in my team, you feel left out unless you compete with each other” and “my team members try to determine how their peers are being evaluated.” The Cronbach’s alpha coefficient for this scale in this context was 0.905.

### 3.3 Control variables

In line with previous studies (Tang et al., 2022; Mu et al., 2023), this study controlled for demographic variables such as employee gender (0 = male, 1 = female); age (1 = 25 years old or younger, 2 = 25–35 years old, 3 = 35–45 years old, 4 = 45 years old or older); level of education (1 = college or associate’ s degree or lower, 2 = bachelor’ s degree, 3 = master’ s degree or higher); and workplace tenure (1 = 1 year or younger, 2 = 1–5 years, 3 = 5–10 years, 4 = 10–15 years, 5 = 15 years or more). In addition, although AI technology has been widely used in various industries (Xu et al., 2020; Gesk and Leyer, 2022; Abbas et al., 2024), the extent of employees’ understanding and perception of AI may differ. This study controlled for industry type by including three dummy variables: technology industry (1 = technology, 0 = otherwise), education industry (1 = education, 0 = otherwise), and service industry (1 = service, 0 = otherwise). The other industries category was used as the reference group.

### 3.4 Statistical analysis

To test the hypotheses proposed in this study, a series of statistical analyses were conducted in a sequential manner.

First, to ensure the reliability of the findings, we assessed potential common method bias and evaluated the validity of the measurement model. Confirmatory factor analysis (CFI) was performed using AMOS 20.0 (Kline, 2011). Convergent validity was established by examining composite reliability (CR) and average variance extracted (AVE), with thresholds set at CR > 0.7 and AVE > 0.5 (Fornell and Larcker, 1981). Discriminant validity was verified using the Fornell–Larcker criterion, whereby the square root of each construct’s AVE must exceed its correlation with any other construct. Model fit was evaluated using multiple indices:χ^2^/df (acceptable if ≤ 5.0; Marôco, 2014), RMSEA (acceptable if ≤ 0.08; Hu and Bentler, 1999), and GFI, NFI, CFI, and IFI (acceptable if ≥ 0.90; Schweizer, 2010). In addition, common method bias was assessed using Harman’s single-factor test in SPSS 22.0 (Tehseen et al., 2017). A significant common method bias was considered present if a single factor explained more than 40% of the total variance (Podsakoff et al., 2003).

Second, descriptive statistics—including means, standard deviations, and Pearson correlation coefficients—were computed for all study variables (i.e., TL, POS, AI-U, CWC) as well as demographic and work-related control variables (gender, age, education level, tenure, and industry) using SPSS 22.0.

Third, hierarchical regression analysis was employed to test the hypothesized relationships (Cohen et al., 2003). All predictor variables were grand-mean-centered to mitigate multicollinearity (Matsunaga, 2008). Path analysis was conducted to examine the structural relationships among TL, POS, AI-U, and CWC. The standardized regression coefficients (β) were interpreted as follows: 0.10 = small effect, 0.30 = medium effect, and ≥ 0.50 = large effect (Cohen, 2013).

Finally, to test the mediation and moderated mediation hypotheses, we applied a bias-corrected bootstrapping procedure with 5,000 resamples using the PROCESS macro for SPSS (Hayes, 2013). Indirect effects were considered statistically significant if the 95% bias-corrected confidence interval did not include zero. For moderated mediation, conditional indirect effects were evaluated at high and low levels of the moderator (±1 SD from the mean).

## 4 Results

To determine the influence of TL on employee AI-U, we aimed to detect relevant patterns in the data obtained through a multipoint questionnaire survey.

### 4.1 Measurement validity and common method bias

We assessed the validity of the measurement model and potential common method bias. First, convergent validity was confirmed as all constructs exhibited composite reliability (CR) values above 0.7 and average variance extracted (AVE) values above the threshold of 0.5 (Fornell and Larcker, 1981). Specifically, the CR values for TL, CWC, POS, and AI-U were 0.858, 0.896, 0.912, and 0.899, respectively, while their corresponding AVE values were 0.563, 0.602, 0.632, and 0.664. Second, discriminant validity was evaluated using the Fornell-Larcker criterion. The square root of each construct’s AVE was greater than its correlations with all other constructs (see Table 1), providing support for discriminant validity.

**Table 1:** Descriptive and bivariate correlation analyses.

Variables	*M*	*SD*	1	2	3	4	5	6	7	8	9	10	11
1. Gender	1.509	0.500	1										
2. Age	2.455	1.116	−0.036	1									
3. Education	2.309	1.065	0.024	0.204**	1								
4. Workplace tenure	2.990	1.409	−0.031	0.023	0.024	1							
5. Industry-education	0.267	0.443	0.033	−0.022	−0.025	−0.045	1						
6. Industry-technology	0.352	0.478	−0.057	0.053	−0.034	0.064	−0.445**	1					
7. Industry-service	0.230	0.422	0.004	−0.041	0.054	−0.003	−0.330**	−0.404**	1				
8. TL	3.107	0.663	−0.039	−0.110*	−0.113**	−0.041	0.030	−0.039	−0.003	1			
9. CWC	3.458	0.885	0.009	−0.059	−0.001	0.047	0.025	−0.048	0.015	0.380**	1		
10. POS	3.242	0.816	−0.027	0.030	0.080	0.000	0.011	0.042	−0.030	0.361**	−.231**	1	
11. AI-U	3.293	0.747	−0.040	−0.078	−0.022	−0.025	0.041	0.005	−0.039	597**	−0.058	459**	1

*n* = 525. Values shown on the diagonal indicate reliability *p < 0.05, **p < 0.01. TL, Transformational Leadership; POS, Perceived Organizational Support; AI-U, Artificial Intelligence Usage. CWC, Competitive Workplace Climate. Source(s): Authors.

Third, common method bias was assessed. The results of a series of competing measurement models demonstrated that the hypothesized four-factor model provided an excellent fit to the data (x^2^/df = 1.648, CFI = 0.973, NFI = 0.934, IFI = 0.973, RMSEA = 0.035), which was superior to all alternative models (see Table 2). The poor fit of the single-factor model (χ^2^/df = 15.11, CFI = 0.394, NFI = 0.38, IFI = 0.397, RMSEA = 0.164, Δχ^2^ = 2823.429, Δdf = 1) further indicates that common method variance is not a serious concern (Podsakoff et al., 2003). This conclusion was reinforced by Harman’s single-factor test, where the first factor accounted for only 25.45% of the variance, well below the 40% threshold (Podsakoff et al., 2003).

**Table 2:** Model fit summary for the hypothesized model and alternative models.

Model	χ^2^/df	RMSEA	CFI	NFI	IFI	GFI	Model comparison test		
							Model comparison	Δχ2	Δdf
The hypothesized four-factor model M1: TL, POS, AI-U, and CWC	1.648	0.035	0.973	0.934	0.973	0.945	--	--	--
The alternative three-factor model M2: TL + POS = 1 factor, AI-U, CWC	7.732	0.113	0.715	0.687	0.716	0.65	M1 VS M2	1258.294***	3
The alternative two-factor model M3: TL + POS + CWC = 1 factor, AI-U	13.468	0.154	0.467	0.45	0.469	0.532	M1 VS M3	2466.739***	2
The alternative one-factor model M4: TL + POS + AI-U + CWC = 1 factor	15.11	0.164	0.394	0.38	0.397	0.466	M1 VS M4	2823.429***	1

*N* = 525. The alternative model was compared with the hypothesized four-factor model. TL, Transformational Leadership; POS, Perceived Organizational Support; AI-U, Artificial Intelligence Usage; CWC, Competitive Workplace Climate. Source(s): Authors.

In summary, the measurement model demonstrates adequate validity, and common method bias is unlikely to be a serious confounding factor in interpreting the relationships among the constructs in this study.

### 4.2 Descriptive statistics and correlations

Descriptive analyses, which were conducted via SPSS 22.0, were based on the mean (*M*) and standard deviation (*SD*). Preliminary analyses were performed to test the relationships between the predictor TL and the outcome variable AI-U. The means (*M*), standard deviations (*SD*), and bivariate correlations for all of the variables are summarized in Table 1. The results of an independent-sample t test indicated that the participants’ demographic characteristics (gender, age, education, workplace tenure, and industry) did not affect AI-U. Pearson’s correlation analysis was used to explore the bivariate associations among the measured variables, and a *p* value < 0.05 was defined as indicating significance. As presented in Table 1, TL was positively related to the dependent variable, that is, AI-U (r = 0.597**, *p* < 0.01), as well as the mediator, i.e., POS (r = 0.361**, *p* < 0.01); in turn, POS was also revealed to be linked to AI-U (r = 0.459**, *p* < 0.01). The moderator variable, i.e., CWC, was negatively related to POS (r = − 0.231 **, *p* < 0.01).

### 4.3 Hypothesis testing

Hierarchical regression analysis was employed to test our hypotheses through a series of nested models. The results of our hypothesis testing are reported in Table 3.

**Table 3:** Hierarchical regression analysis results regarding the effect of TL on employee AI-U.

Variables	AI-U																			
	Model 1				Model 2				Model 3				Model 4				Model 5			
	β	SE	t	*p*	β	SE	t	*p*	β	SE	t	*p*	β	SE	t	*p*	β	SE	t	*p*
Constant	-	0.175	20.265	0.000	-	0.200	5.781	0.000	-	0.191	8.530	0.000	-	0.357	−4.658	*0.000*	-	0.453	0.728	0.467
Gender	−0.044	0.065	−0.998	0.319	−0.018	0.053	−0.508	0.612	−0.011	0.049	−0.323	0.747	−0.002	0.044	−0.075	0.940	−0.003	0.042	−0.118	0.906
Age	−0.079	0.030	−1.773	0.077	−0.026	0.024	−0.708	0.480	−0.034	0.022	−1.022	0.307	−0.034	0.020	−1.128	0.260	−0.037	0.019	−1.257	0.209
Education	−0.001	0.031	−0.027	0.978	0.055	0.025	1.513	0.131	0.070	0.023	2.087	0.037	0.038	0.021	1.232	0.219	0.032	0.021	1.081	0.280
Tenure	−0.024	0.023	−0.546	0.585	−0.003	0.019	−0.088	0.930	0.019	0.017	0.578	0.564	0.020	0.016	0.675	0.500	0.013	0.015	0.464	0.643
Industry-education	0.038	0.105	0.617	0.538	0.040	0.085	0.790	0.430	0.040	0.078	0.870	0.385	0.024	0.071	0.580	0.562	0.022	0.068	0.549	0.583
Industry-technology	0.016	0.101	0.242	0.809	0.044	0.081	0.842	0.400	0.033	0.075	0.685	0.494	0.024	0.068	0.543	0.587	0.010	0.065	0.245	0.806
Industry-service	−0.023	0.108	−0.376	0.707	−0.010	0.087	−0.210	0.834	−0.010	0.080	−0.228	0.820	−0.019	0.073	−0.458	0.647	−0.014	0.070	−0.364	0.716
TL					0.600	0.040	16.826	0.000	0.329	0.040	8.225	0.000	0.847	0.125	6.766	0.000	0.735	0.221	3.741	0.000
CWC													0.803	0.095	7.123	0.000	−0.166	0.152	−0.921	0.275
CWC*TL																	−0.108	0.059	−0.342	0.733
POS									0.536	0.030	17.867	0.000					0.365	0.050	6.740	0.000
R^2^	0.011				0.361				0.457				0.554				0.590			
Adjusted R^2^	−0.002				0.352				0.448				0.545				0.581			
F	*F* (7,517) = 0.828, *p* = 0.565				*F* (8,516) = 36.508, *p* = 0.000				*F* (9,515) = 48.239, *p* = 0.000				*F* (10,514) = 63.813, *p* = 0.000				*F* (11,513) = 67.157, *p* = 0.000			
ΔR^2^	0.011				0.350				0.096				0.096				0.036			
ΔF	F (7,517) = 0.828, *p* = 0.565				*F* (1,516) = 283.114, *p* = 0.000				*F* (1,515) = 91.090, *p* = 0.000				*F* (1,514) = 111.135, *p* = 0.000				*F* (1,513) = 45.434, *p* = 0.000			

Variables	POS															
	Model 6				Model 7				Model 8				Model 9			
	β	SE	t	*p*	β	SE	t	*p*	β	SE	t	*p*	β	SE	t	*p*
Constant	-	0.191	16.150	0.000	-	0.271	10.612	0.000	-	0.271	12.273	0.000	-	0.305	−19.564	0.000
Gender	−0.027	0.072	−0.610	0.542	−0.025	0.072	−0.563	0.573	−0.018	0.069	−0.433	0.665	0.003	0.038	0.133	0.894
Age	0.010	0.033	0.218	0.828	0.014	0.033	0.311	0.756	0.007	0.032	0.153	0.878	0.006	0.017	0.273	0.785
Education	0.082	0.034	1.830	0.068	0.086	0.034	1.920	0.055	0.099	0.033	2.287	0.023	0.016	0.018	0.679	0.497
Tenure	−0.005	0.025	−0.111	0.911	−0.003	0.025	−0.074	0.941	0.016	0.025	0.370	0.712	0.019	0.013	0.804	0.422
Industry-education	0.047	0.115	0.745	0.456	0.047	0.115	0.747	0.455	0.047	0.111	0.784	0.434	0.006	0.061	0.187	0.852
Industry-technology	0.068	0.110	1.047	0.296	0.070	0.110	1.081	0.280	0.060	0.106	0.970	0.332	0.037	0.058	1.079	0.281
Industry-service	0.009	0.118	0.143	0.886	0.010	0.118	0.159	0.874	0.010	0.114	0.165	0.869	−0.012	0.062	−0.383	0.702
TL					0.509	0.057	8.93	0.001	0.147	0.054	2.721	0.007	0.304	0.107	2.47	0.004
CWC									−0.290	0.042	−6.331	0.000	−0.265	0.081	3.275	0.000
CWC*TL													−0.315	0.029	−10.862	0.000
POS																
*R^2^*	0.011				0.013				0.084				0.728			
Adjusted R^2^	−0.003				−0.002				0.068				0.722			
F	F (7,517) = 0.798, *p* = 0.589				F (8,516) = 0.841, *p* = 0.567				F (9,515) = 5.257, *p* = 0.000				F (10,514) = 137.350, *p* = 0.000			
ΔR^2^	0.011				0.002				0.071				0.644			
ΔF	F (7,517) = 0.798, *p* = 0.589				F (1,516) = 1.136, *p* = 0.287				F (1,515) = 40.081, *p* = 0.000				F (1,514) = 1214.679, *p* = 0.000			

*n* = 525. TL, Transformational Leadership; POS, Perceived Organizational Support; AI-U, Artificial Intelligence Usage; CWC, Competitive Workplace Climate. Source(s): Authors.

Hypothesis 1 proposed that TL positively enhances employee AI-U in the workplace. As indicated in Model 2 in Table 3, TL was positively related to employee AI-U (*β =* 0.600***, SE = 0.040, *p* < 0.001). Additionally, the SEM strategy based on maximum likelihood estimation that was employed with the assistance of AMOS 20.0 indicated that the model exhibited a good fit to the data: χ^2^/df = 2.12, GFI = 0.973, NFI = 0.952, IFI = 0.974, TLI = 0.966, CFI = 0.974, RMSEA = 0.046. Therefore, Hypothesis 1 was supported.

Hypothesis 2a proposed that TL positively impacts employee POS. As showed in Model 7, TL was positively related to POS (*β =* 0.509***, SE = 0.057, *p* < 0.001). Therefore, Hypothesis 2a was supported.

Hypothesis 2b proposed that POS positively impacts employee AI-U. As indicated in Model 3, POS was positively related to AI-U (*β* = 0.536***, SE = 0.030, p < 0.001); thus, Hypothesis 2b was supported.

Hypothesis 2c proposed that POS positively mediates the relationship between TL and AI-U. Compared to the effect in Model 2 (*β* = 0.600), the direct positive effect of TL on AI-U in Model 3 was attenuated but still significant (*β* = 0.329***, SE = 0.040, p < 0.001). This finding indicated that POS partly mediated the relationship between TL and AI-U. Furthermore, bootstrap analysis revealed that the indirect effect of TL on AI-U via POS was significant (*β* = 0.273**, p < 0.01; SE = 0.064, t = 4.266, 95% CI = [0.148, 0.398], excluding 0), and the mediating effect (TL → POS → AIU) accounted for 45.5% of the total effect; the tested model exhibited good fit indices (χ^2^/df = 1.657, GFI = 0.961, NFI = 0.949, IFI = 0.979, TLI = 0.974, CFI = 0.978, RMSEA = 0.036). Thus, hypothesis 2c was supported.

Hypothesis 3a proposed that CWC moderates the relationship between TL and AI-U. As indicated in Model 5 in Table 3, the ability of the interaction term of CWC * TL to predict employee AI-U was nonsignificant (*β* = −0.108, *p* > 0.05); thus, Hypothesis 3a was not supported. This non-significant finding is itself informative. The near-zero coefficient suggests that the positive influence of TL on AI-U may be stable, regardless of the level of CWC. This could be because transformational leaders effectively mitigate the potential stressors of competition by fostering a shared vision, thereby consistently encouraging AI-U.

**Figure 2:**
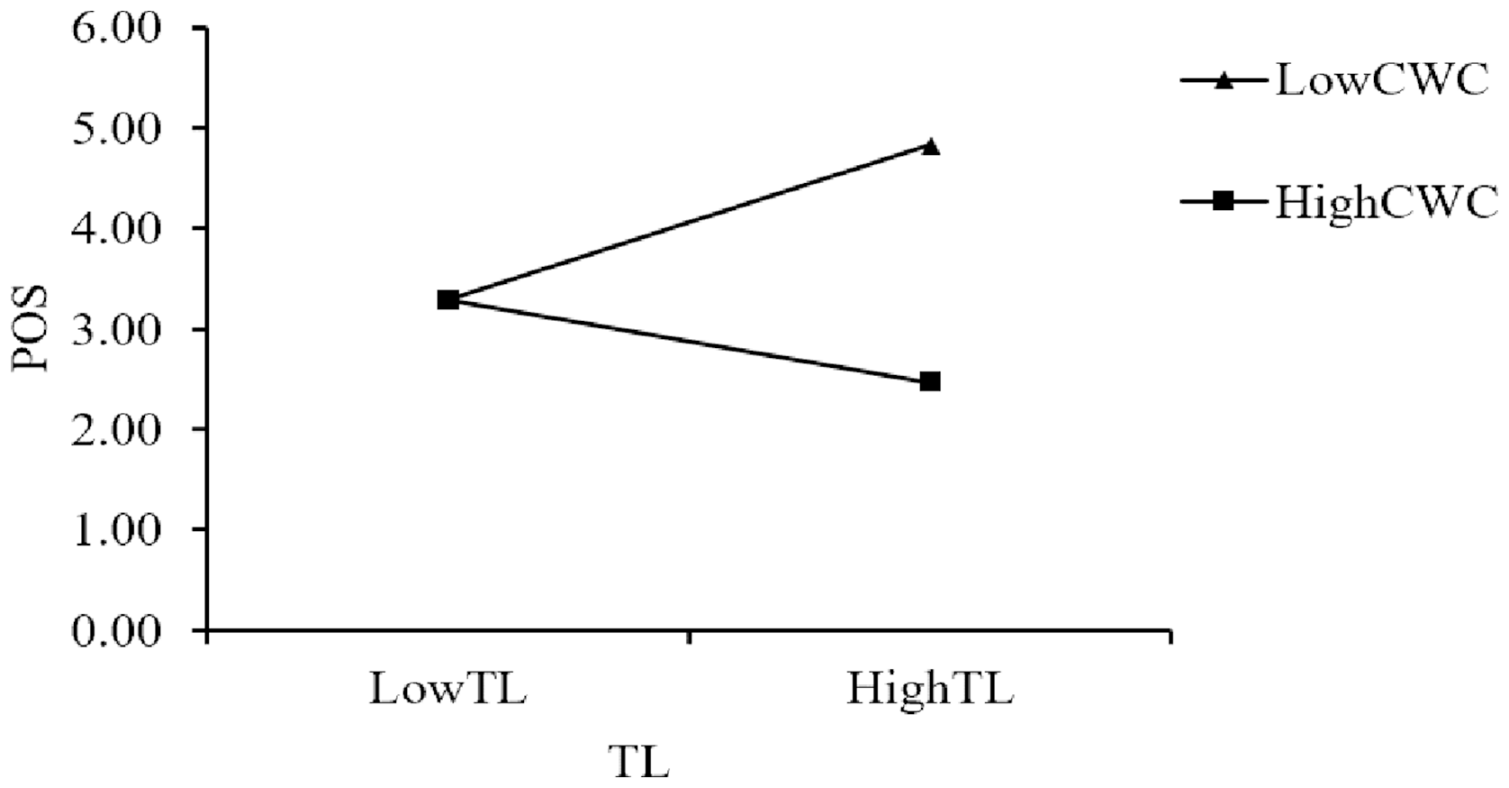
The moderating effect of CWC on the relationship between TL and POS.

Hypothesis 3b predicted that the positive relationship between TL and POS is stronger when CWC is low (vs. high). As indicated in Model 9 in Table 3, the ability of the interaction term of CWC *TL to predict POS was significant (*β* = −0.315***, SE = 0.029, *p* < 0.001). The simple slope analysis confirmed this pattern. As shown in Figure 2, the effect of TL on POS was strongest when CWC was at a low level (*β* = 0.775, SE = 0.038, t = 20.103, *p* < 0.001, 95% CI = [0.629, 0.921], excluding 0). This effect remained significant but weakened as CWC increased, to *β* = 0.757 (SE = 0.053, t = 14.015, *p* < 0.001, 95% CI = [0.683, 0.831], excluding 0) at the mean level of CWC, and to *β* = 0.739 (SE = 0.075, t = 10.394, *p* < 0.001, 95% CI = [0.636, 0.843], excluding 0) at a high level of CWC. Although the absolute difference in the simple slopes was modest, the consistent decreasing pattern supports the hypothesis. Thus, Hypothesis 3b was supported.

Hypothesis 3c predicts that CWC negatively moderates the indirect effect of TL on AI-U via POS. To test these moderated mediation hypotheses, the bias-corrected bootstrap analysis method provided the most accurate confidence interval (CI) estimation for the indirect effect at both high (1 SD above) and low (1 SD below) levels of the moderator. As indicated in Table 4, the indirect effect was significant when CWC was low (*β* = 0.393, SE = 0.064, 95% CI = [0.274, 0.522] excluding 0), whereas the indirect effect was also significant but was significantly weaker when CWC was high (*β* = 0.207, SE = 0.034, 95% CI = [0.140, 0.274], excluding 0). Additionally, the index of moderated mediation was −0.186 (SE = 0.053, 95% CI = [−0.290, −0.082], excluding 0). The moderating model containing the independent (TL), dependent (AI-U), and moderator (CWC) variables alongside the interaction variables (TL*CWC) exhibited an acceptable fit to good fit (χ^2^/df = 1.698, GFI = 0.935, NFI = 0.924, TLI = 0.970, CFI = 0.964, RMSEA = 0.032). Thus, Hypothesis 3c was supported.

**Table 4:** The moderated mediating effects.

Moderator	TL → POS → AI-U			
CWC	Effect	SE	BootLLCI	BootULCI
Low (-SD)	0.393	0.064	0.274	0.522
High (+SD)	0.207	0.034	0.140	0.274
Deviance	−0.186	0.053	−0.290	−0.082

TL, Transformational Leadership; POS, Perceived Organizational Support; AI-U, Artificial Intelligence Usage; CWC, Competitive Workplace Climate, Source(s): Authors.

## 5 Discussion

AI usage (AI-U) in the workplace is becoming increasingly prevalent. While previous studies have focused predominantly on the influence of individual-level characteristics on employee AI-U (Park and Woo, 2022; Potinteu et al., 2023), other factors particularly leadership styles have received comparatively less attention. Based on SCT, this study examined the effect of TL on employee AI-U, with particular attention to the mediating role of perceived organizational support (POS) and the moderating role of competitive workplace climate (CWC). Our findings offer clear support for the proposed hypotheses:

H1, which posited a positive effect of TL on AI-U, was supported.
H2, predicting the mediating role of POS in the relationship between TL and AI-U, was also supported, with POS shown to partially mediate this effect.
H3, which proposed that POS mediates the relationship between TL and AI-U, and that this indirect effect is negatively moderated by CWC, was supported. However, it proposed that CWC positively moderates the positive relationship between TL and AI-U, was not supported.

Overall, this study helps elucidate the psychological mechanisms through which TL influences employee AI-U and addresses a significant gap in the AI-U literature by incorporating leadership and climate variables.

### 5.1 Theoretical implications

Our findings have several important theoretical implications.

First, grounded in social cognitive theory (SCT), this study extends prior findings on the antecedents of employee AI-U by identifying TL style as a key environmental factor. Existing research has predominantly emphasized individual-level characteristics (e.g., personalities and perceptions) as significant predictors of AI-U (Park and Woo, 2022; Potinteu et al., 2023; Mousavi et al., 2025). While these studies highlight the role of personal factors in shaping usage behavior, SCT posits that human behavior emerges from the dynamic interplay between personal factors, environmental influences, and behavior itself (Bandura, 1986; Schunk and DiBenedetto, 2020). Within organizational settings, leaders represent a critical environmental component that shapes observational learning and behavioral modeling (Bandura, 1986). Although prior work has acknowledged leadership’s general impact on employee attitudes toward technology adoption (Peifer et al., 2022; He et al., 2023; Liu et al., 2024), scant attention has been paid to how specific leadership styles, particularly TL, affect AI-U. By empirically demonstrating the significant role of TL, this study addresses the research gap concerning this topic and providing a new perspective for research on employee AI-U and the application of SCT.

Second, this study has discussed the mediating role of POS in the impact of TL on employee AI-U. Previous studies have focused primarily on the direct relationships between individual characteristics and employee AI-U (Park and Woo, 2022; Potinteu et al., 2023), thereby neglecting the potential mediating effects of employees’ inner psychological perceptions, which are shaped by the external setting. The research reveals that POS can mediate the relationship between TL and employee AI-U. Specifically, the findings suggest that TL can enhance employee POS, thus increasing employee AI-U extent in the workplace. This is in line with previous research that has highlighted the positive impact of TL on employees’ attitudes and behavior (Bakker et al., 2023; Qalati et al., 2022). Furthermore, by identifying POS as a key mediator, this research elucidates the socio-cognitive pathway through which TL influences employee AI-U. This finding advances the understanding of AI-U beyond purely instrumental or technological models, repositioning it as a socially embedded behavior shaped by employees’ perceptions of organizational backing.

Third, and most critically, this study elucidates the nuanced role of contextual factors by examining the moderating effect of the CWC. A pivotal finding is the stability of the direct effect of TL on employee AI-U across varying levels of CWC, indicating a non-significant moderating effect. This finding suggests that the motivational influence of TL on specific, task-relevant behaviors is stable enough to transcend immediate competitive team dynamics. Employees likely perceive adopting leader-endorsed technologies as a direct response to supervisory expectations, an response that appears impervious to the intensity of peer competition. This resilience emphasizes the stability of the direct, instrumental reliance mechanism, which seems less vulnerable to the social comparisons inherent in CWC than the socio-cognitive pathway involving POS. In contrast to the null moderation on the direct effect, our results demonstrate that CWC significantly weakened the positive indirect effect of TL on AI-U through POS. This differential pattern clarifies the specific boundary condition of CWC’s influence. It operates not by blunting the direct motivational link from leadership but by selectively eroding the sense of organizational support that facilitates indirect influence. This finding empirically validates the dual-mechanism framework proposed in our hypothesis development, demonstrating that the mechanism of instrumental reliance can sustain the direct TL-AI-U link even as the mechanism of eroded organizational support undermines the mediating role of POS. Furthermore, this finding underscores the importance of distinguishing between leadership’s direct motivational influence on behavior and its indirect effect through socio-cognitive mechanisms. Our results reveal that these pathways exhibit differential sensitivities to the same contextual factor. This advances social cognitive theory (SCT) by demonstrating that the “social environment” can selectively influence cognitive assessments (e.g., POS) more than direct behavioral links in the leadership process. Consequently, this study contributes to the broader literature on CWC by moving beyond a simplistic “good or bad” dichotomy (Murtza and Rasheed, 2023; Wang et al., 2024) and instead clarifying the specific pathways through which competition exerts its complex influence within organizational dynamics.

### 5.2 Management implications

The results of our research have several important practical implications for organizations. First, the significant positive effect of TL on AI-U demonstrates leadership as a critical enabler of technological adaptation. Organizations may therefore consider integrating transformational competencies (e.g., inspirational communication and change management) into leadership selection criteria and development programs, particularly for roles central to digital transformation efforts. Second, the mediating role of perceived organizational support (POS) indicates that employees’ adoption of AI stems not merely from top-down advocacy but from a genuine sense of organizational backing. Consequently, leadership development should extend beyond technical instruction on AI applications to emphasize how leaders can foster a supportive climate. Training programs could equip leaders to actively demonstrate concern for employee needs and provide adequate resources, thereby strengthening POS as the psychological pathway facilitating voluntary AI uptake. Third, the moderated mediation analysis reveals that the indirect effect of TL on AI-U via POS is attenuated in highly CWC. This suggests that CWC can undermine the supportive mechanisms through which TL promotes AI-U. For managers, this finding highlights an important risk: although competitive pressures may compel superficial compliance with AI-U, they are unlikely to foster the sustained, value-based engagement necessary for long-term effectiveness. Organizations should therefore be cautious about cultivating intensely competitive environments when seeking to promote voluntary, extra-role behaviors such as proactive AI-U If competition is necessary, it is advisable to carefully design its implementation to avoid eroding employees’ sense of support, perhaps by complementing TL with strong organizational justice and transparent reward systems.

### 5.3 Limitations and directions for future research

Although this study offers valuable theoretical and practical contributions, it is not without limitations.

First, the research design used in this study is cross-sectional. Although the findings align with theoretical expectations, the potential for causal relationships to evolve over time—due to shifts in individual perceptions—cannot be entirely excluded. Future studies could account for several covariates closely associated with AI-U—such as employees’ prior AI experience and technology-related education—as well as job characteristics, including job complexity and task-level AI dependency, within a hierarchical linear modeling framework. Furthermore, to more rigorously establish temporal precedence and validate the causal pathways proposed in the model, future research could employ a three-wave longitudinal design conducted over a 12-month period.

Second, although our sample encompassed a range of industries, its heterogeneity may introduce unintended variability in organizational factors such as culture, leadership styles, and the maturity of AI implementation. We have attempted to mitigate this concern by statistically controlling for industry type in our analyses. However, we acknowledge that unmeasured industry-specific may still influence the findings and potentially affect their interpretability. Subsequent studies would include industry-specific factors (e.g., finance, healthcare vs. Tech; nuanced organizational policies or cultural norms) and the maturity of organizational AI governance policies as control or moderating variables to better isolate the unique effect of leadership and avoid confounds. Moreover, the concentration of our sample within the technology, education, and service sectors (accounting for 85% of participants) suggests that our findings may be most readily applicable to knowledge-intensive and service-oriented environments. Future studies would therefore benefit from employing stratified sampling strategies to ensure a more balanced representation across industries or, conducting in-depth, industry-specific investigations to elucidate the contextual boundaries of our model. In addition, the reliance on the WJX platform may constrain the sample’s representativeness. To address this limitation, future studies should adopt mixed-methods sampling strategies that combine online and offline recruitment channels, such as community-based recruitment and multi-platform collaboration.

Third, given our explicit focus on sanctioned AI usage (AI-U), this study conceptualizes employee AI-U as a behavior occurring within an organizational context that formally approves and implements AI tools. However, it is important to acknowledge that employees may still engage in uses that are discouraged or explicitly prohibited, even when the organization generally supports AI adoption. Future research should thus distinguish more finely between “sanctioned” and “unsanctioned” AI usage. Such a distinction could reveal, for example, whether a competitive climate exerts opposing effects—promoting unsanctioned use for short-term gains while undermining sanctioned use that relies on organizational resources. Furthermore, although the scale used in this study was contextualized within examples of sanctioned AI-U, the items themselves remain general in phrasing. It would be valuable for subsequent studies to develop behavior-specific scales that explicitly differentiate between sanctioned and unsanctioned usage, enabling a more nuanced understanding of their respective antecedents and outcomes.

Fourth, employees’ attitudes and behaviors toward AI are shaped by the interplay of multiple factors at both the individual and environmental levels. Therefore, to further elucidate the mechanisms underlying these attitudes and behaviors, future research may incorporate a broader range of variables that may influence employees’ AI-U. Given that AI-U is fundamentally human-driven, individual differences—such as personality traits, inner motivation, risk tolerance, and familiarity with AI technologies—may play critical roles in shaping employee AI-U and should be examined in subsequent studies. Moreover, prior studies suggests that leaders play a critical role in AI adoption decisions, and the successful AI implementation AI hinges on their acceptance and active support (Peifer et al., 2022; He et al., 2023; Liu et al., 2024; Mousavi et al., 2025). However, leader beliefs about AI are not a monolith (Mahmud et al., 2023; Mousavi et al., 2025), they may hold favorable or unfavorable attitudes toward AI. So the future research would consider leaders’ individual attitudes towards AI (e.g., AI anxiety, perceived utility), which in turn affects employee AI-U. Furthermore, the relationships between TL, POS, and AI-U are likely not universal but are instead moderated by contextual factors. These may include internal elements such as organizational culture and team dynamics, as well as external pressures like environmental uncertainty and broader cultural values. To test the boundary conditions and generalizability of our model, a avenue for future research involves direct cross-cultural comparisons. For example, a study could contrast how these relationships operate in individualistic cultures (e.g., the U.S.) versus collectivistic cultures (e.g., South Korea, Chinese). Such a design would not only test the model’s stability but also elucidate how cultural norms shape the psychological processes underlying employee AI adoption.

## Data Availability

The original contributions presented in the study are included in the article/supplementary material, further inquiries can be directed to the corresponding author.

## Footnotes

^1^ https://www.wjx.cn/
